# Proscription supports robust perceptual integration by suppression in human visual cortex

**DOI:** 10.1038/s41467-018-03400-y

**Published:** 2018-04-17

**Authors:** Reuben Rideaux, Andrew E. Welchman

**Affiliations:** Department of Psychology, University of Cambridge, Downing Street, Cambridge, CB2 3EB UK

## Abstract

Perception relies on integrating information within and between the senses, but how does the brain decide which pieces of information should be integrated and which kept separate? Here we demonstrate how proscription can be used to solve this problem: certain neurons respond best to unrealistic combinations of features to provide ‘what not’ information that drives suppression of unlikely perceptual interpretations. First, we present a model that captures both improved perception when signals are consistent (and thus should be integrated) and robust estimation when signals are conflicting. Second, we test for signatures of proscription in the human brain. We show that concentrations of inhibitory neurotransmitter GABA in a brain region intricately involved in integrating cues (V3B/KO) correlate with robust integration. Finally, we show that perturbing excitation/inhibition impairs integration. These results highlight the role of proscription in robust perception and demonstrate the functional purpose of ‘what not’ sensors in supporting sensory estimation.

## Abstract (web summary)

Perception relies on information integration but it is unclear how the brain decides which information to integrate and which to keep separate. Here, the authors develop and test a biologically inspired model of cue-integration, implicating a key role for GABAergic proscription in robust perception.

## Introduction

Our impression of the surrounding world is built upon fragmentary sensory information that is always incomplete and often ambiguous. To achieve perception, the brain combines a range of signals subject to different constraints. For instance, judging the shape of a nearby object may rely on fusing information from different visual cues (e.g., perspective, shading, and texture) and modalities (e.g., size from vision and touch)^1^. By integrating signals, observers resolve ambiguities and judgments become more precise^2^.

Understanding of integration has generally focused on the performance benefits that result from combination^3^. In particular, psychophysical work has shown that participants’ precision improves near-optimally when integrating cues, closely matching the expectations of maximum likelihood estimation^4^. However, if cues originate from different objects, or specify different things, it no longer makes sense to integrate them. A new pair of glasses, for example, can suddenly mean that trusted cues (such as binocular disparity and texture) specify conflicting shapes^5^. If the brain nevertheless persisted in averaging the information together, observers could perceive something incompatible with either cue, leading to errors (e.g., reaching to the wrong location). This process of dealing with conflicting signals and deciding whether or not to integrate them has been described as one of causal inference^6^, and presenting such stimuli provides an ideal testbed for probing the mechanisms that underlie perceptual integration. Behavioural evidence^7^ suggests that integration degrades gracefully under cue conflict^8^. However, we have little understanding of how this is achieved by the human brain: neither in theory nor in practice.

Here we develop and test a biologically inspired model of integration that captures improved performance when information is consistent, yet, unlike previous models, shows robust behaviour in the face of conflict. We propose a role for proscription in optimal sensory encoding and suggest that it makes sense for the brain to employ ‘what not’ detectors, i.e., neurons selective for stimuli that do not correspond to real objects. These units facilitate robust sensory estimation by driving suppression of unlikely interpretations of the local environment. We have recently provided evidence for this principle in encoding binocular disparity^9^; here we demonstrate its utility in a new model that combines different depth cues for robust shape perception.

The central premise of our model is that the brain uses what not detectors that respond best to discrepancies between two cues. These responses are useful because they increase suppression of certain perceptual interpretations. To test for neurobiological correlates of this process, we examined the relationship between suppressive processing in the human brain and perceptual integration. We focused on a region of the dorsal visual cortex (area V3B/KO) that is intricately involved in integrating three-dimensional (3D) cues to object shape^10–13^. Under the proscriptive model, we hypothesised that suppressive processing in this region is associated with robust integration.

We indexed suppression using non-invasive magnetic resonance spectroscopy (MRS) measures of the inhibitory neurotransmitter γ-aminobutyric acid (GABA)^14–16^. We tested whether robust integration relates to GABA concentration around V3B/KO. We then used transcranial direct current stimulation (tDCS) to perturb the excitatory/inhibitory balance of underlying cortical tissue. We tested whether disrupting processing in this way would lead to reduced perceptual integration. We show that GABA concentrations around area V3B/KO correlate strongly with robust perceptual cue integration, and that tDCS applied over V3B/KO leads to impaired integration.

In line with the proscriptive model, our empirical results demonstrate the critical role of suppressive signals in shaping integration of 3D cues to object perception. Using detectors that respond to unrealistic combinations of features makes sense theoretically and has correlates with suppressive processing in the human brain. Finally, we show that the proscriptive framework provides a natural link to phenomena of rivalry and perceptual bistability^12,17–19^. Studies of alternating perceptual states have provided a useful tool to access conscious experience, but have been long divorced from models of routine perceptual processing. Our work shows that such phenomena reflect the operation of a generalized mechanism for sensory processing that exploits what not signals to effect perception.

## Results

### Robust integration of depth cues

**Fig. 1:**
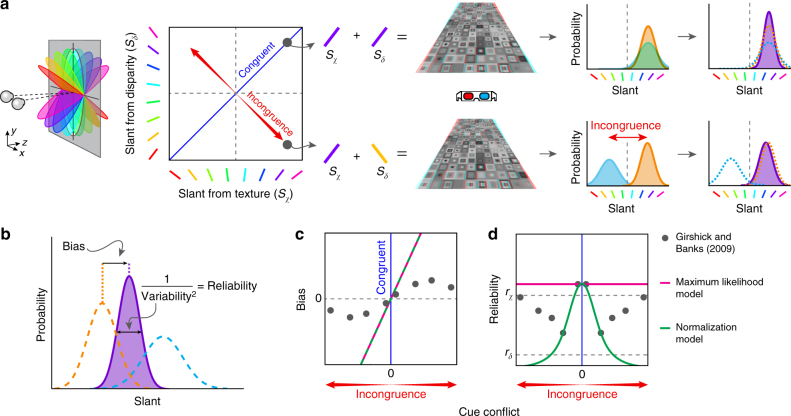
Illustrations of cue incongruence and the performance of previous models relative to psychophysical data. **a** The space of possible slants depicted by texture and disparity cues. To illustrate cue integration we provide a cartoon of the slant of 3D surfaces defined by congruent (top) and incongruent (bottom) cues for viewing through red–cyan anaglyph glasses. Sensory estimates are modelled as Gaussian distributions. For congruent cues (top row), fusion (purple curve) produces a more reliable estimate (i.e., lower variance) than for either component (orange, blue curves). For incongruent cues (bottom row), accuracy is maintained by down-weighting the influence of the less reliable cue. **b** Illustration of the bias (offset in the mean) and reliability (inverse of the variance) parameters used to describe the data. **c**, **d** Behavioural measures extracted from Girshick and Banks^7^ showing **c** perceived slant (quantified by bias away from the more reliable cue) and **d** reliability as a function of cue conflict. Pink and green curves indicate the predictions made by the maximum likelihood model and the Normalization model^25^, respectively

Human perception is typically robust in the face of discrepant information. To understand how this could be achieved by the brain, it is useful to start by thinking about the space of possible viewed stimuli defined by combinations of two 3D-shape cues (binocular disparity *S*_*δ*_ and texture *S*_*χ*_, Fig. 1a). Any stimuli falling along the positive diagonal in this space specify exactly the same shape from the two cues—i.e., the information is congruent (*S*_*δ*_ = *S*_*χ*_). Similar to previous work^7,20–22^, we can independently manipulate the cues to explore the effects on perception: moving away from the positive diagonal increases the degree of incongruence between the cues. By systematically manipulating incongruence, Girshick and Banks^7^ found that perception is initially biased away from the more reliable of the two cues, but then returns to the more reliable cue as conflict increases (Fig. 1b, c).

This graceful behaviour is also found for changes in the variability associated with participants’ judgments: with no conflict, the reliability increases above that of either component in line with the optimal integration of the signals^3^. As conflict increases, however, reliability falls and then recovers to that associated with the more reliable component (Fig. 1d), where this relationship can be described using a second-order Gaussian derivative function. If the conflict between cues increases still further, there can be complete scission, and eventually rivalry between alternative perceptual interpretations^22,23^. How is this behaviour implemented by the brain?

The standard mechanistic account of perceptual integration is described as a process of maximum likelihood estimation^8,24^. This model uses information provided by the cues optimally, such that judgments are more reliable than is possible on the basis of either cue alone (Fig. 1b). A biologically plausible model of integration^25^ can capture the improved performance associated with combined cues using a population of neurons. However, neither model captures the robustness of human perceptual performance (Fig. 1c, d, pink and green lines). Here we propose a model that shows improved reliability when signals are consistent, but down-weights the influence of the less reliable cue in cases of incongruence.

### Derivation of the proscriptive integration model

To implement a biologically plausible model of robust cue integration, we consider the estimation of surface slant—a key percetual quantity that underlies multiple behaviours—using binocular disparity and texture depth cues. In a significant departure from previous work^25^, we use model neurons (‘units’) that respond best to incongruent cues (*S*_*δ*_ ≠ *S*_*χ*_) to incorporate suppressive computations into the model. This allows the model to produce robust perceptual estimates that exceed single cues for congruent information, but revert to the most reliable source of information in the face of discrepancy.

**Fig. 2:**
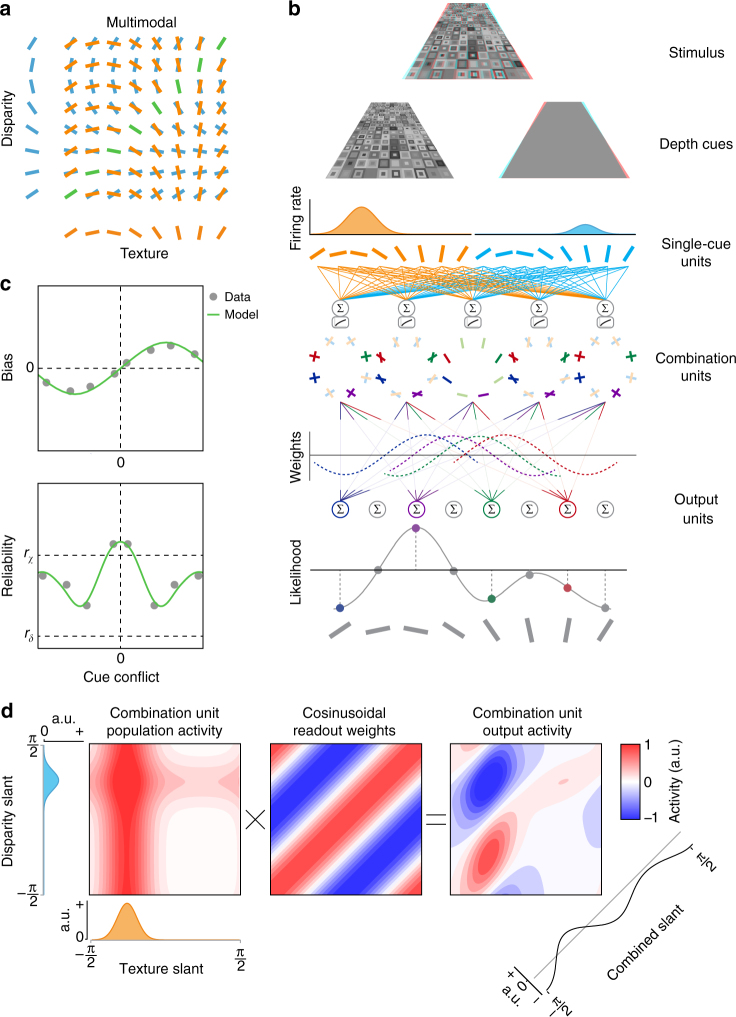
Schematic illustration of the proscriptive integration model of cue integration. **a** Cartoon of the network’s detectors. The input layer of the model consists of unimodal detectors for slant defined by disparity or texture. These are represented along the ordinate and abscissa. The next layer of the model is the combination units, which are tuned to different combinations of unimodal inputs. **b** Overview of the model’s architecture. A slanted 3D Voronoi stimulus (illustrated for red–cyan anaglyph glasses) activates separate unimodal units tuned to disparity or texture. The response of unimodal units is pooled by combination units. The activity of combination units is then read out according to a cosinusoidal weighting function to produce a likelihood function of slant angle. **c** Bias and reliability as a function of cue conflict, generated by the proscriptive integration model using experimental data from Girshick and Banks^7^. **d** Example simulation of the model when presented with conflicting cues. Combination unit activity is equal to the sum of unimodal (texture/disparity) responses. This response is multiplied by a cosine weight matrix, representing readout weights to the output layer. The final estimate is produced by summing diagonally

The front end of the model consists of a bank of filters that encode the slant of the surface from a single cue: one set for disparity and another for texture. A layer of combination units then integrates signals from the two cues (Fig. 2a). It is noteworthy that the majority of the combination units are incongruent, i.e., best stimulated by a discrepancy between the information provided by each cue. Although this may appear counterintuitive, there is evidence that such neurons exist in the primate brain^26^, although the functional significance of these units has hitherto been opaque.

Based on empirical evidence^27^, we assume that combination units perform a sum of their inputs that increases monotonically, but sublinearly, with stimulus intensity (Fig. 2b). This nonlinearity models sublinear response functions of the combination units, which could be mediated by means of synaptic depression or normalization^28^. We generated responses for each unit independently according to a Poisson distribution with a mean firing rate given by the tuning curves (see the ‘Proscriptive integration model’ section in Methods).

Following single-cue input, combination units are read out by a layer of output units, where readout weights are defined by a cosine function. Simulating a range of cue conflicts produces a pattern of robust estimates consistent with empirical observations^7^ (Fig. 2c). In particular, when cues are consistent, or nearly so, the model predicts performance that is better (i.e., higher reliability) than either cue alone, with a maximum reliability equal to the quadratic sum of the component cues, in line with optimal estimation for consistent cues^1,4^. As incongruence increases, the reliability falls below that of the more reliable cue (*χ*) and bias increases above zero. However, increasing incongruence still further produces a robust reversion to *χ* in terms of both estimator bias and reliability.

To illustrate the model computations, consider a stimulus that indicates incongruent slants from the two cues (Fig. 2d). We express the initial activity in the combination layer as their summed responses in disparity–texture space. Values along the diagonal represent the activity of congruent units and values off the diagonal represent the activity of incongruent units. By multiplying this activity by a cosine weight matrix, a map of activity is produced, which shows the influence of each unit on the final output. The sum of activity along the diagonal yields the final slant estimate. After converting activity to firing rate by thresholding negative values to zero, estimator reliability is derived from the height of the peak^29^. We quantify bias as the difference in slant angle between the final estimate and the more reliable of the two cues. The input cue reliability is derived from the height of the peak produced in the absence of additional cues.

The critical feature of the model is the form of the readout weights, which we implement as a basic cosine function. The only additional parameter models tonic inhibition within the model, which we assumed to be 5% below equilibrium^30–32^. The intutition behind the model is that when two cues are present a maximum likelihood process indicates that the best evidence for an estimate is in between the two cues. However, the location where the evidence is strongest is not always a realistic interpretation, i.e., when cues are conflicting. Thus, as conflict between cues increases, activation in the cosinusoidal weight matrix turns from positive to negative, penalizing the midpoint between cue estimates. By penalizing the midpoint in cases of conflict, the evidence now maximally supports the estimate of the more reliable cue.

### Behavioural measures of robust perception

**Fig. 3:**
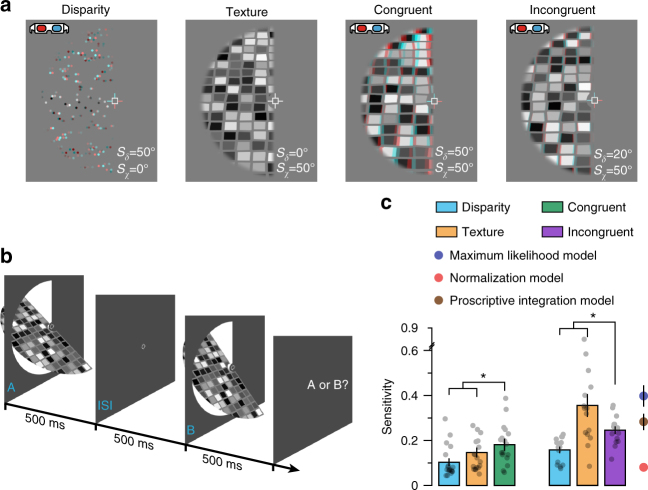
Psychophysical measurements of cue integration. **a** Single- and (congruent/incongruent) combined-cue stimuli designed for use with red–cyan anaglyphs. **b** Presentation sequence used to psychophysically assess cue integration: observers judged whether the first or second stimulus was more slanted. **c** Behavioural sensitivity (the reciprocal of the just noticeable difference in degrees) for slant defined by disparity, texture, and congruent and incongruent combinations of these cues. We assessed the statistical significance in congruent and incongruent conditions using RM ANOVAs. In both cases we found significant main effects of stimulus type (congruent: *F*_2,34_ = 7.92, *P* = 0.002; incongruent: *F*_2,28_ = 10.68, *P* = 0.002). We followed this with paired *t*-tests to compare individual stimulus conditions; asterisks highlight significant differences *P* < 0.05 (congruent disparity: *t*_18_ = 3.85, *P* = 0.001; congruent texture: *t*_18_ = 2.54, *P *= 0.02; incongruent disparity: *t*_14_ = 3.58, *P* = 0.003; incongruent texture: *t*_14_ = 2.16, *P* = 0.048). Performance in the single-cue conditions was used to generate predictions of incongruent-cue sensitivity based on the maximum likelihood model, the normalization model^25^ and our proscriptive integration model. As explained in the Procedure section of the Methods, using different slant angles was necessary for the congruent and incongruent stimulus conditions; in particular, to ensure robust behaviour, sensitivity had to be much higher for one cue than the other. This accounts for the differences in sensitivity observed between single cues in congruent (left three bars) vs. incongruent (right three bars) stimulus conditions. To ensure that reduced sensitivity for incongruent cues (relative to texture) was not due to using a different slant angle, we confirmed that, as expected, sensitivity was also better for congruent cues at higher slant angles. Semi-transparent black dots indicate individual data points and error bars indicate SEM

To test the predictions of our model, we assessed how sensitive participants were in discriminating the slant of a viewed object (Fig. 3a, b), measuring the just noticeable difference (j.n.d.) threshold between two stimuli to provide a measure of perceptual reliability. We manipulated the stimuli such that they differed in only one cue (i.e., single-cue changes in disparity or texture), or the slant specified by the texture and disparity cues was consistent, or inconsistent. To maximize the chances of observing robust behaviour, we designed the incongruent stimuli such that the texture cue (mean sensitivity, 0.35) was approximately twice as reliable as the disparity cue (mean sensitivity, 0.16).

As expected, when participants viewed stimuli with congruent cues, behavioural sensitivity was significantly higher than for the single-cue conditions (disparity, *t*_17_ = 3.85, *P *= 0.001, Cohen’s *d* = 0.91; texture, *t*_17_ = 2.54, *P* = 0.02, Cohen’s *d* = 0.60; Fig. 3c). However, given that the three models we compare are inseparable for congruent stimuli, the important comparison is the case when participants viewed incongruent-cue stimuli. We found that performance was significantly worse than for single-cue slant defined by texture (*t*_14_ = 2.16, *P* = 0.048, Cohen’s *d* = 0.51), but significantly better than that for single-cue slant defined by disparity (*t*_14_ = 3.58, *P *= 0.003, Cohen’s *d* = 0.84). This shows that the presence of the less reliable cue impaired the participants’ perceptual estimates, but that the falloff in performance was not completely catastrophic in that it remained above that of the less reliable cue. A possible concern might be that the texture cue was perceived at a smaller slant than specified^33^, resulting in the congruent-cue stimuli being perceived as incongruent, and vice versa. However, the sensitivity in these conditions, relative to that for single cues, confirms that this was not the case. That is, congruent-cue sensitivity is higher than that for either single cue and incongruent-cue sensitivity is not, as would be expected if the cues that comprise the stimuli in these conditions were congruent and incongruent, respectively. Further, observers’ estimates were significantly biased towards the slant defined by the texture cue in the incongruent condition (*t*_17_ = 29.03, *P* = 5.4e^–16^, Cohen’s *d* = 6.84), consistent with robust estimation for incongruent cues. Using the sensitivities measured for single cues, we generated predictions for sensitivity in the incongruent-cue condition based on (i) the maximum likelihood model, (ii) Ohshiro et al.^25^ normalization model and (iii) our proscriptive integration model. Comparing Bayes factor scores, we found that the proscriptive integration model best accounted for incongruent-cue sensitivity, 14.2 times better than the maximum likelihood model and 4.3e^5^ times better than the normalization model (Fig. 3c).

### Relating perceptual judgments to suppressive processing

**Fig. 4:**
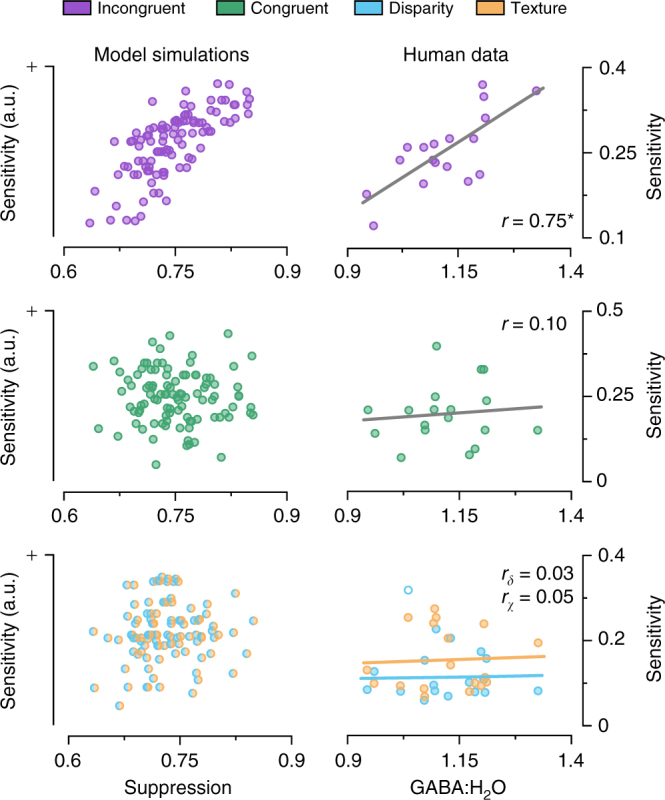
Model predictions and empirical measurements on the relationship between behavioural sensitivity and the level of suppression. The left column shows proscriptive integration model simulation results that manipulated the inhibitory gain in the model: more suppression leads to higher model sensitivity for incongruent cues (top row), but not congruent (middle row) or single cues (bottom). The right column shows the results of MRS measurements that quantified GABA around V3B/KO and related this to between-subject differences in behavioural sensitivity. Consistent with the model simulations, we found a significant positive correlation between human observers’ GABA concentration in the voxel centred on V3B/KO and perceptual sensitivity to incongruent-cue stimuli (*n* = 18, Pearson’s correlation *r* = 0.75, *P* = 3.8e^−4^, CI_95%_ = [0.40, 0.91]; confidence intervals are corrected for multiple comparisons; top right), but not to congruent- or single-cue stimuli (*n* = 18, congruent: Pearson’s *r* = 0.10, *P* = 0.68, middle right; disparity: Pearson’s *r* = 0.03, *P* = 0.92; texture: Pearson’s *r* = 0.05, *P* = 0.85; bottom right). Asterisks highlight significant differences *P* < 0.05. Each datum represents measures from one participant; bivariate outliers are shown as empty circles

The proscriptive integration model posits a central role for suppression to infer the probable structure of the local environment. To demonstrate this, we simulated performance in response to single, congruent and incongruent stimuli, while varying the magnitude of the model’s negative (i.e., suppressive) readout weights (Fig. 4, left). We found no relationship between suppression and the model’s sensitivity to single or congruent cues (*n* = 100, single/congruent: Pearson’s *r* = 0.14, *P* = 0.16). However, for incongruent stimuli, there was a strong positive correlation between the model’s suppressive weights and sensitivity (*n* = 100, Pearson’s *r* = 0.78, *P* = 1.1e^−21^). (This pattern of results persisted under changes in the simulation parameters, Supplementary Figure 1).

Experimentally, we reasoned that differences between human observers in their sensitivity to incongruent cues might relate to differences in suppressive tone within the cortex. In particular, we employed MRS that has previously been used to link neurochemistry to visual processing^14,16^. We tested for correlations between (at rest) concentrations of the main inhibitory neurotransmitter (GABA) within the participants’ brains and robust perceptual judgements measured using psychophysics.

We considered three regions of interest in the participants’ brains. Our primary interest was in an MRS voxel centred on area V3B/KO of the human brain as previous functional magnetic resonance imaging (fMRI) work demonstrated that this area is intricately involved in cue integration^10–13^. In addition, we measured control voxels in the visual (V1) and motor (M1) cortices. We anticipated that greater potential for suppression (as indexed by overall GABA levels) would be associated with robust perceptual judgments for regions of the cortex associated with cue integration. Consistent with this prediction, we found a significant positive correlation between GABA concentration in the voxel centred on V3B/KO and perceptual sensitivity to incongruent stimuli (*n* = 18, Pearson’s *r* = 0.75, *P* = 3.8e^–4^; CI_95%_ = [0.40, 0.91]; confidence intervals are corrected for multiple comparisons; Fig. 4, top right).

In line with the model predictions (Fig. 4, left), we found that the relationship between GABA concentrations and behavioural performance was specific to the incongruent stimuli. That is, we found no relationship between GABA and slant defined by disparity, texture, or congruent cues (*n* = 18, disparity [30°,20°]: Pearson’s *r* = 0.03,– 0.3, *P* = 0.92,0.26; texture [30°,50°]: Pearson’s *r* = 0.05,– 0.49, *P* = 0.85,0.08; congruent: Pearson’s *r* = 0.10, *P* = 0.68; Fig. 4, right; Supplementary Figure 2b). Importantly, the relationship between GABA and behavioural performance was specific to the V3B/KO region: we found no relationship between sensitivity and GABA measured over the V1 and M1 control sites (*n* = 14, V1: Pearson’s *r* = 0.40, *P* = 0.15; *n* = 18, M1: Pearson’s *r* = – 0.21, *P* = 0.39; Supplementary Figure 2a).

In addition to sensitivity, we also used the measures of bias in the incongruent-cue simulations to calculate the weights assigned by the model to each cue. As with sensitivity, suppression in the model was highly correlated with the weight given to the more reliable cue (*n* = 100, Pearson’s *r* = 0.78, *P* = 1.7e^−21^; Supplementary Figure 2c). We found the equivalent relationship between GABA concentration in humans from a voxel centred on V3B/KO and the weight (i.e., normalized bias) they assigned to the more reliable cue (*n* = 12, Pearson’s *r* = 0.60, *P* = 0.04; Supplementary Figure 2c). Its is noteworthy that the observed reliable-cue weights (mean = 0.53) were smaller than that predicted by the model (mean = 0.87; Supplementary Figure 2c). A plausible explanation for this is that for the incongruent-cue reference stimulus, the texture cue was more influenced by a frontoparallel bias, whereas this was not the case for the congruent-cue test stimuli^34^. Thus, although the cues were perceived as incongruent, the magnitude of this difference was reduced as a result of a small frontoparallel bias acting on the texture cue.

As GABA concentration is expressed with reference to H_2_O, a possible concern might be that the observed correlations with behavioural performance relate to individual variability in H_2_O concentration rather than GABA. However, we found the same result when GABA was referenced to Creatine (Supplementary Figure 3a). Although this is reassuring, as an additional check, we also quantified the concentration of Glx (glutamate and glutamine) in the spectra and tested whether this correlated with behaviour when referenced to H_2_O. We found no evidence of a relationship between V3B/KO Glx:H_2_O concentration and robust behavioural performance (Supplementary Figure 3b), indicating that individual variability in H_2_O concentration did not explain the results. Another possible concern is that the observed correlations relate to the ratio of grey matter (GM) and white matter (WM) content in the voxels. However, there was no relationship between incongruent-cue sensitivity and GM:WM voxel content (Supplementary Figure 3c), and the relationship between incongruent-cue sensivity and GABA remained significant after controlling for GM:WM voxel content (*n* = 18, Pearson’s *r* = 0.63, *P* = 0.007).

### Perturbing processing to alter perception of combined cues

To move beyond correlative evidence, we next sought to perturb the excitatory-inhibitory balance of the cortex, and then measure the consequences on perceptual judgments. To this end, we applied tDCS to perturb cortical excitability centred over area V3B/KO in 12 human participants. This technique has previously been shown to alter overall responsivity of the visual cortex^35,36^ and produce systematic effects on visual judgments^37^. We applied anodal and cathodal stimulation montages to V3B/KO before measuring sensitivity to single-, congruent-, and incongruent-cue stimuli. (Given the complexity of the technique^38^, we had no a priori expectation about the effects of tDCS polarity, other than noting previous work had seen stronger effects of cathodal stimulation in the visual cortex^35,39^.) To control for placebo effects, we contrasted the results with sensitivity following sham stimulation. We reasoned that if tDCS is targeting the process of robust integration (consistent with a V3B/KO locus) single-cue performance should be relatively unaffected, as this information is extracted earlier in the cortical hierarchy to the stage of combination. It should be noted that information from other cues is also present in the single-cue conditions^20,21^ (e.g., the random dots of the sterogram provide a texture cue). However, as this information is (i) less reliable than the dominant cue (e.g., dots provide only small texture elements) and (ii) common to the pairs of stimuli being judged, it is likely to have little influence on perception. In contrast, combining two reliable cues (in the combined-cue conditions) results in large perceptual changes, so disrupting integration in these conditions will produce a larger effect.

**Fig. 5:**
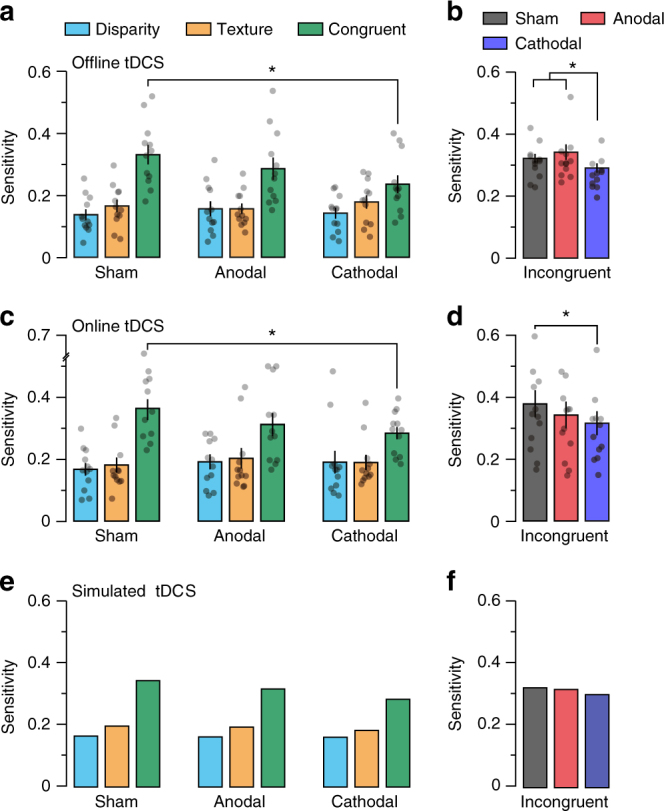
Behavioural and model simulated effects of tDCS on single-(texture/disparity), congruent- and incongruent-cue conditions. **a**, **b** Perceptual sensitivity for slant defined by **a** single (texture/disparity), congruent and **b** incongruent cues following 20 min sham, anodal, and cathodal stimulation targeting V3B/KO. **c**, **d** Same as **a** and **b** but during stimulation. RM ANOVA was used to test for main effects across experimental conditions. In **a** there was a significant main effect of stimulus type (*F*_2,22_ = 23.25, *P* = 3.7e^−6^) and a significant interaction (*F*_4,44_ = 4.40, *P* = 0.004). In **b** a significant main effect of stimulation (*F*_2,22_ = 4.76, *P* = 0.02) was followed by paired *t*-tests: sham vs. cathodal conditions for congruent (*t*_11_ = 5.17, *P* = 3.0e^−4^, Cohen’s *d* = 1.49) and incongruent cues (*t*_11_ = 2.58, *P* = 0.02, Cohen’s *d* = 0.74). **c**, **d** We tested the reproducibility of these effects and found significant differences between sham vs. cathodal conditions for congruent (*t*_11_ = 2.61, *P* = 0.02, Cohen’s *d* = 0.75) and incongruent cues (*t*_11_ = 2.46, *P* = 0.03, Cohen’s *d* = 0.71). Asterisks highlight significant differences *P* < 0.05. Semi-transparent black dots indicate individual data points and error bars indicate SEM. To test whether these effects could be captured by the model, we first simulated performance in the sham conditions of the **a**–**d** tDCS experiments (**e**, **f**, sham conditions). We then simulated anodal and cathodal stimulation by fitting two additional free parameters using the tDCS effects measured in the single- and congruent-cue conditions (**e**, anodal/cathodal conditions); these parameters model the effects of tDCS on inhibition and excitation. Finally, we simulated performance for incongruent cues (based on the fixed parameters) (**f**, anodal/cathodal conditions). The model predictions match the mean experimental data

In line with our reasoning, we found that sensitivity in the disparity- and texture- single-cue conditions was unaffected by the application of tDCS (disparity anodal: *t*_11_ = 1.32, *P* = 0.21; disparity cathodal: *t*_11_ = 0.58, *P* = 0.58; texture anodal: *t*_11_ = 0.63, *P* = 0.54; texture cathodal: *t*_11_ = 1.08, *P* = 0.30; Fig. 5a). In the sham condition, we observed the expected behavioural benefit from combination: i.e., performance in the congruent-cue condition was significantly higher than for the single-cue conditions (disparity, *t*_11_ = 7.57, *P* = 1.1e^−5^, Cohen’s *d* = 2.18; texture, *t*_11_ = 5.67, *P* = 1.4e^−4^, Cohen’s *d* = 1.63).

Importantly, however, the benefit of combining cues in the congruent- and incongruent-cue conditions was reduced through the application of tDCS. In particular, we found that cathodal tDCS reduced sensitivity to both congruent and incongruent cues (congruent, *t*_11_ = 5.17, *P* = 3.0e^−4^, Cohen’s *d* = 1.49; incongruent, *t*_11_ = 2.58, *P* = 0.02, Cohen’s *d* = 0.74), whereas lower performance under anodal stimulation was not statistically significant (congruent, *t*_11_ = 1.33, *P *= 0.21; incongruent, *t*_11_ = 0.56, *P *= 0.61; Fig. 5a, b). This indicates that perturbing the cortical excitability around V3B/KO disrupted observers’ ability to integrate disparity and texture cues, while leaving sensitivity to the individual cues unaffected.

tDCS modulates the excitability of neural tissue during stimulation (online effects) and following the offset of stimulation (offline effects)^35^. Although the modulatory effects of on- and offline tDCS are similar, pharmacological work indicates that the neurophysiological mechanism that produces this modulation may be different^40^. We therefore tested whether cue integration is also impaired by online tDCS, repeating the experiment in a new set of participants, and now measuring behavioural performance during stimulation. We found the same pattern of results: sensitivity to combined congruent/incongruent cues was significantly reduced during cathodal tDCS (congruent, *t*_11_ = 2.69, *P* = 0.02, Cohen’s *d* = 0.78; incongruent, *t*_11_ = 2.98, *P *= 0.01, Cohen’s *d* = 0.86; Fig. 5c, d). These results show that cue integration is impaired by both on- and offline tDCS, and provide a replication of the main tDCS effects on a second cohort of participants.

To assess whether tDCS affected observers’ bias, we tested for differences in the point of subjective equality between stimulation conditions. We found marginally significant effects (repeated measures analysis of variance (RM ANOVA), offline: *F*_2,22_ = 3.22, *P* = 0.06; online: *F*_2,22_ = 2.72, *P* = 0.09); however, the differences were small and the opposite direction for on- vs. offline stimulation. Moreover, the largest difference between on- and offline stimulation is between the sham conditions that provides the control baseline (Supplementary Figure 4). We therefore interpret these results as serendipitous.

The population-level neurophysiological impact of tDCS is not yet understood in sufficient detail to permit its effects to be accurately simulated within our model. Instead, we took the more realistic approach of testing how well the model could capture the tDCS results by using a subset of experimental conditions to fix the model’s parameters, and then tested for generalization to other conditions. We first simulated performance in the sham conditions so that it fit the experimental data (difference between simulated and experimental data, disparity: *t*_23_ = 0.70, *P* = 0.49; texture: *t*_23_ = −1.65, *P* = 0.11; congruent: *t*_23_ = 0.33, *P* = 0.74; incongruent: *t*_23_ = 0.75, *P* = 0.46; Fig. 5e, f). Having determined the main properties of the model, we then introduced two additional free parameters to capture the effects of tDCS. These parameters independently multiplied the strength of the positive and negative weights in the combination layer by a factor between zero and one, modelling the (at least partially) independent effects of tDCS on inhibition and excitation^41^. We fit these parameters using the tDCS effects measured in the single- and congruent-cue conditions (Fig. 5e). Finally, we simulated performance for incongruent cues (based on the fixed parameters) and compared the model’s predictions with the empirical data. We found that the simulated tDCS results fit the observed data well: anodal effects were meagre, reflecting the nonsignificant changes observed, whereas cathodal effects were equivalent in direction and magnitude to those observed (difference between simulated and experimental effect of cathodal stimulation, congruent: *t*_23_ = 1.63, *P* = 0.12; incongruent: *t*_23_ = 1.90, *P* = 0.07; Fig. 5f). The difference between cathodal and anodal stimulation is consistent with previous work showing that cathodal stimulation has a greater effect on visual evoked potentials^35,39^.

We built a number of controls into our tDCS protocol to rule out alternative interpretations of our findings. First, we used lateralized stimulus presentation, with some stimuli presented in the visual hemifield that would be affected by tDCS and others in the non-stimulated hemifield. We found that the effects of stimulation over V3B/KO were spatially localized to the visual hemifield contralateral to target stimulation, that is, there was no effect of stimulation on performance for stimuli presented ipsilaterally (RM ANOVA, *F*_1,35_ = 1.19, *P* = 0.31; Supplementary Figure 5a). To assess the regional specificity of the tDCS, we tested the effects of tDCS using a V1 tDCS montage, and found no effect of tDCS (i.e., no difference in congruent-cue sensitivity between sham and cathodal stimulation; *t*_11_ = 0.20, *P* = 0.84; Supplementary Figure 6). This suggests that stimulation over V3B/KO can produce specific effects on cue integration in line with previous fMRI work that highlighted the role of this area in depth cue integration^10–13^. Another possible concern is that stimulation could produce a nonspecific effect by reducing general behavioural performance (e.g., through distraction caused by skin irritation). Our results made this unlikely as we found that tDCS had a specific effect on integration conditions and not on single cues. However, we had included trials in the experimental design to act as ‘lapse’ tests under the different stimulation conditions. Specially, we presented some easy trials for which performance should be close to 100% correct; we found no evidence for a change in general performance resulting from stimulation (RM ANOVA, *F*_1,35_ = 2.40, *P* = 0.11; Supplementary Figure 5b), indicating that tDCS effects were task specific. We also found no effect of stimulation on response times (RM ANOVA, *F*_2,22_ = 0.69, *P* = 0.49; Supplementary Figure 5c). Finally, measuring binocular eye movements during tDCS showed that stimulation did not disrupt eye movement control (critical for stereopsis): eye vergence and version was stable and not systematically affected by stimulation (Supplementary Figure 7).

### Modelling perceptual rivalry

Having considered imaging evidence consistent with the proscriptive model, we make a final observation about its utility for understanding other perceptual phenomena. If conflicting or ambiguous images are presented to a viewer, such as in the case of binocular rivalry or viewing a Neckar cube, viewers typically experience perceptual alternation over time. Traditionally, the study of such perceptions has been kept quite separate from models of routine perceptual processing^42,43^. By contrast, here show that proscription provides a natural foundation that accommodates both routine perceptual estimation and alternating perceptual interpretations.

**Fig. 6:**
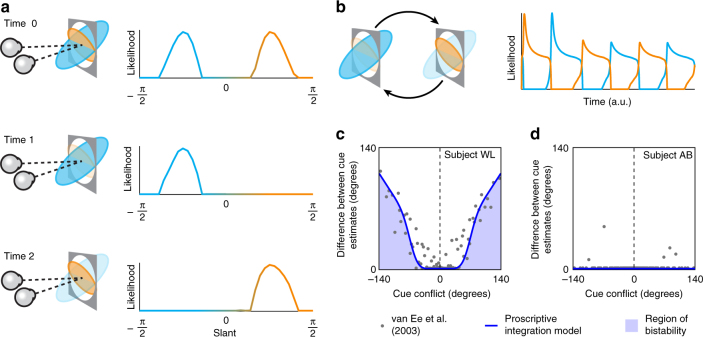
Simulating perceptual rivalry with the proscriptive integration model. **a** Perceptual rivalry produced with the model: time 0 indicates the likelihood function represented in the output layer when two incongruent cues of equal reliability are simulated. Mutual inhibition between neuronal representations (and internal noise) results in dominance at time 1. Adaptation of the units representing the dominant representation results in its gradual decay and subsequent re-emergence of the non-dominant representation at time 2. **b** Likelihood of the simulated slant angles as a function of time, showing the typical dynamics of perceptual alternation. **c** Psychophysical data extracted from van Ee et al.^23^. This shows the difference between cue estimates as a function of cue conflict (see Online Methods for detailed description of data extraction and reanalysis). The blue line is a fit of the proscriptive model and the shaded area indicates the experience of perceptual rivalry. **d** Depicts an example subject from van Ee et al.^23^ in which one cue dominates perception and rivalry is not experienced. The blue line (barely visible) shows the fit of the proscriptive model where the two cues are ascribed very different reliabilites such that one completely dominates the other

Up to this point, we have considered robust perception under conflict when one cue is considerably more reliable than the other. We now consider the case where cues are in conflict but equally reliable. In this instance, there is no principled way of selecting one cue over the other and the result is typically bistable perception^22,23^. The proscriptive integration model naturally accommodates this behaviour: when conflicting cues are simulated with similar reliability, a bimodal population response is observed in the output (Fig. 6a, time 0).

We can model perceptual alternation by assuming a combination of mutual inhibition and adaptation between the competing, bimodal representations^19,42^. The dynamics of adaptation allow us to simulate the temporal dynamics of perceptual bistability (Fig. 6b). Specifically, mutual inhibition between neuronal representations (and internal noise) initially results in the dominance of one of the cues over the other (Fig. 6a, time 1). However, through adaptation, the activity of units representing the dominant representation gradually decays and is followed by the re-emergence of the non-dominant representation (Fig. 6a, time 2). This cycle forms the basis of the rate of swapping between perceptual estimates (Fig. 6b).

Given the mechanism of mutual inhibition and adaptation is well-established, it is perhaps unsurprising that it can be used to produce bistability here. However, using this mechanism to extend the proscriptive integration model, we were able to capture the behaviour of observers viewing conflicting cues to slant measured by a previous study^23^. In particular, we simulated a range of cue conflicts and assessed the degree of bistability in the model’s estimates. This allowed us to capture human psychophysical performance that shows the situations in which bistability is experienced (Fig. 6c, shaded blue region). The previous work also reported cases in which participants did not experience any bistability (Fig. 6d). We captured this within the model on the basis that such observers ascribe unequal reliabilities to the presented cues, such that one cue always dominates.

## Discussion

To understand the structure of the surrounding world, the brain integrates information from a range of sensory cues. Integration can improve perceptual estimates; however, it needs to be sensitive to the context: in some cases it is better to down-weight some signals.This process of deciding whether or not to integrate cues has been described as one of causal inference^6^, that is, inferring whether multiple sensory signals were produced by the same or different sources. Here we demonstrate a principled way in which this can be done. In particular, we develop a model whose central premise is that certain sensory signals are used proscriptively to drive suppression of particular interpretations and thereby facilitate robust integration. We demonstrate that proscription drives both (a) robust integration and (b) perceptual rivalry. Further, we provide evidence for neural correlates of proscription in the human brain. In particular, we find that (i) GABA measured in a brain region intricately involved in cue fusion (V3B/KO) is strongly correlated with robust perceptual integration and (ii) perturbing the excitatory/inhibitory balance with tDCS impairs perceptual integration.

Our prosrciptive model demonstrates why it makes sense for the brain to employ what not detectors that respond best to stimuli that do not correspond to the features of real objects. By so doing, these detectors drive suppression of unlikely interpretations of the local environment. Although this may appear counterintuitive, there is evidence that what not neurons exist in the primate brain, although their functional purpose was previously unclear.

Previous electrophysiological recordings have shown that although certain neurons are tuned to the same information specified by two cues (congruent neurons), many others respond best when there is a large conflict between the information provided by two cues (incongruent neurons)^26,44,45^. Why should the brain develop such neurons? One possibility is that they are used as a veto^46^. Here we demonstrate that incongruent signals provide a key means of supporting robust integration: a single model explains cases when cues are combined to boost performance, when discrepant signals are down-weighted and cases of complete scission.

Our formulation also provides an architecture for processes of recalibration that are likely to constitute an important facet of perceptual integration. In particular, a change in the observer’s state, such as wearing a new pair of glasses, or sustaining an injury, can necessitate that the information provided by two cues is recalibrated. A neural architecture that is specialized only for congruent signals requires a recalibration of the individual sensory estimates. However, within our model, recalibration could be achieved by simply changing the phase of the readout weight matrix (see Parise and Ernst^47^ for a similar example).

For simplicity, in this study we have only considered the inclination of a surface away from frontoparallel (slant), whereas real-world surfaces are typically parameterized as the combination of slant and tilt (orientation of the surface in the image plane). Extending our model to accomodate both slant and tilt should be feasible within the suggested architecture, simply necessitating an increase in the number of units to accommodate joint encoding of slant and tilt^48^.

A central premise of the model is that incongruent neurons are used proscriptively to drive suppression of unlikely perceptual interpretations. In support of this, we identify neural correlates of suppression that predict robust perceptual behaviour. Specifically, we find baseline inhibitory neurotransmitter GABA concentration is correlated with robust perceptual estimates. Moreover, we find that the GABA associations were regionally specific to cortical areas associated with depth cue integration (V3B/KO); we find no correlation between robust perception and GABA measured at control regions (V1 and M1).

The application of MRS in humans has started to provide new insight into perceptual and cognitive processes^14,16^; however, a known limitation of the technique is its spatial resolution. Although we centred data acquisition on particular brain regions (e.g., V3B/KO), the size of the voxels necessary for the technique (3 × 3 × 2 cm) inevitably means that we sampled from neighbouring regions of the cortex (e.g., V3A, V3, and V7). With this in mind, we selected the locations of our control sites to demonstrate a level of regional specificity. Moreover, extensive fMRI work has identified V3B/KO as a locus for depth cue integration^10–13^, supporting the interpretation that GABA measured in this area was the primary contributor to the relationship with robust integration.

Another limitation of MRS is that it measures total concentration of neurochemicals within a localized region and cannot distinguish between intracellular and extracellular pools of GABA. This is relevant, because these pools are thought to have different roles in neuronal function. Here we show that the suppressive gain of the network is correlated with robust perception, suggesting that the relationship between MRS-measured GABA and robust perceptual behaviour is, at least partially, correlated with intracellular vesicular GABA, which drives neurotransmission^49^. However, the tonic cortical inhibition incorporated within the proscriptive integration model, which is maintained by extracellular GABA^50^, also facilitates robust estimates. Thus, the correlation between MRS-measured GABA and robust perception may also be driven by extracellular GABA.

Our results show that GABA is linked to the robust selection that occurs when two cues are in conflict and one is perceived as more reliable. We also show that the proscriptive model can reproduce behaviour when conflicting-cue stimuli are presented that result in perceptual rivalry^22,23^. We therefore envisage robust selection and perceptual rivalry as falling on a continuum of degrees of cue conflict, which is moderated by the relative reliability between cues. Within this framework, it makes sense that GABA concentrations have been linked to the perception of bistable stimuli. Specifically, the rate of swapping between bistable stimuli is correlated with GABA concentration in human visual cortex^16,51^. Moreover, previous theoretical and empirical work indicates that incongruent neurons may have a key role in perceptual rivalry^19,52^. Here we propose proscription as a common mechanism (operating across a range of cue conflicts) to support robust integration by driving suppression of unlikely interpretations of the local environment.

Having discovered suppressive correlates of robust perceptual integration, we perturbed the cortical excitability around V3B/KO using tDCS. We found that following cathodal stimulation, estimates produced by both congruent and incongruent cues were impaired, whereas anodal stimulation produced a smaller, nonsignificant effect. We also demonstrate that these effects replicate in an independent sample of participants. Importantly, we showed that these effects were specific to the process of integrating cues rather than the processing of single cues per se. We could capture this behaviour within the proscriptive integration model by fitting two free parameters that attenuated the strength of positive/negative lateral connections within the combination layer to the results for congruent and single cues, and then using these (now fixed) parameters to predict the effect of stimulation on incongruent-cue estimates.

Recent meta-analyses have questioned the reliability of certain tDCS findings. However, tDCS has been shown to reliably change GABA concentrations^41,53^, modulate visual evoked potentials^35,36^ and affect visual perception^37^. The replication of the basic effect in an independent sample of participants is thus important in providing reassurance about the reliability of the findings we report. A principle limitation of tDCS is that its effects are spatially imprecise^54^. To address this limitation, we combined MRI functional localization of V3B/KO, neuronavigation and electric-field simulations to produce a tDCS montage that would most effectively target V3B/KO. Further, we repeated the experiment with a montage targeting V1 and found no effect.

The interaction between the current flow induced by tDCS and the unique morphology of the brain means that the effects of stimulation, even directly under the electrode, are too complex to characterize as either purely increasing or decreasing excitation^38^. With this in mind, here we used tDCS to perturb the balance of excitation and inhibition around V3B/KO, and designed our experiment with a range of controls that allowed us to make precise interpretations of the results. Interestingly, we found no evidence of polarity-specific directional effects, i.e., modulation in one direction for anodal and another for cathodal^35^, yet our results are consistent with evidence that cathodal stimulation is more effective than anodal in modulating the excitability of the visual cortex^35,39^ and may suggest that the morphology of the visual cortex is more amenable to the current produced by cathodal stimulation.

Together, our modelling and empirical results point to a central role for proscription in driving robust perceptual integration. Using neurons that respond to unrealistic combinations of features to drive robust perception makes sense theoretically and has correlates with suppressive processing in the human brain. This suggests a generalized mechanism for sensory processing that exploits what not information to facilitate perception and provides a natural foundation to explain phenomena associated with rivalry and perceptual bistability.

## Methods

### Participants

Observers were recruited from the University of Cambridge and had normal or corrected-to-normal vision, and were screened for stereo deficits. A priori sample sizes were established using effect sizes from previous MRS^14^ and tDCS^35^ studies to achieve 90% power. Twenty observers participated in the MRS experiment; however, two were not included in the analysis: one withdrew mid-scan and a hardware fault stopped acquisition mid-scan for the other. Eighteen subjects (15 male; 17 right-handed; 25.1 ± 3.1 years) completed MRS for V3B/KO and M1, of whom 15 also returned for the (control) V1 scan. Twelve observers participated in each of the 5 tDCS experiments, for a total of 34 different observers (19 male; 31 right-handed; 24 ± 3.6 years). Experiments were approved by the University of Cambridge ethics committee; all observers provided written informed consent.

### Apparatus and stimuli

Stimuli were generated in MATLAB (The MathWorks, Inc., Matick, MA) using Psychophysics Toolbox extensions^55,56^. Binocular presentation was achieved using a pair of Samsung 2233RZ LCD monitors (120 Hz, 1680 × 1050) viewed through mirrors in a Wheatstone stereoscope configuration. The viewing distance was 50 cm and participants’ head position was stabilized using an eye mask, head rest and chin rest. Eye movement was recorded binocularly at 1 kHz using an EyeLink 1000 (SR Research Ltd, Ontario, Canada).

Stimuli were virtual planes slanted about the horizontal axis (Fig. 3a). Two cues to slant were independently manipulated: texture and disparity. The texture cue was generated by Voronoi tessellation of a regular grid of points (1° ± 0.1° point spacing) randomly jittered in two dimensions by up to 0.3°^21,57^. Each texture patch had on average 64 texture elements (textels); however, the actual number of textels varied between trials depending on their size. Each textel was randomly assigned a grey level and shrunk about its centroid by 20%, creating the appearance of ‘cracks’ between textels. The width of these cracks also varied as a function of surface slant, thus providing additional texture information. Texture surfaces were mapped onto a vertical virtual surface and rotated about the horizontal axis by the specific texture-defined angle, before a perspective projection consistent with the physical viewing geometry was applied. To isolate the disparity cue, a random-dot stimulus was generated using the same parameters as in the texture stimuli, i.e., an average of 64 dots with randomized grey level assignment. In the single-cue disparity and two-cue conditions, binocular disparity was calculated from the cyclopean view and applied to each vertex/dot based on the specific disparity-defined slant angle.

Surfaces were presented unilaterally (80% left and 20% right of fixation) inside a half-circle aperture (radius 6°) and a cosine edge profile to blur the appearance of depth edges. Stimuli were presented on mid-grey background, surrounded by a grid of black and white squares (75% density) designed to provide an unambiguous background reference. In the stereoscopic conditions, observers could theoretically discriminate surface slant based only on the difference in depth at the top/bottom of a pair of stimuli. Similarly, in the texture-only condition, observers could make judgements based on the difference in textel density at the top/bottom of a pair of stimuli. To minimize the availability of these cues, disparity-defined position was randomized by shifting the surface relative to the fixation plane (0° disparity) to between ± 10% of the total surface depth. Texture-defined position in depth—which corresponded to average textel size—was randomized for each stimulus presentation by increasing point spacing in the initial grid of points by ± 10%^21^.

We presented four cue conditions: 2× single-cue (texture and disparity) and 2× two-cue conditions (congruent and incongruent). Stimuli in the single-cue texture condition were presented monocularly (right eye), whereas all other stimuli were presented binocularly.

### Procedure

Observers performed a two-interval forced-choice discrimination task in which the reference and test stimuli were presented in randomized order (Fig. 3b). Each stimulus was presented for 500 ms with an inter-stimulus interval of 300 ms. Following the offset of the second stimulus, observers were prompted to indicate which stimulus was more slanted (using a keypress) by the fixation cross changing from white to black. No duration limit was enforced for responses, but observers were encouraged to respond quickly. Following a response, the fixation cross was changed back to white and a fixation period of 500 ms preceded the onset of the next trial. A method of constant stimuli procedure was used to control the difference in slant between the reference and test stimuli. The MATLAB toolbox Psignifit^58^ (http://psignifit.sourceforge.net/) was used to fit psychometric functions to the data. Sensitivity to slant was derived from the slope of the psychometric function and the point of subjective equality (PSE) from the threshold.

In the congruent-cue condition, reference stimuli consisted of consistent texture and disparity slant (*S*_*δ*_ = *S*_*χ*_ = 40°). It is noteworthy that we chose this slant angle, as observers sensitivity to disparity and texture cues was similar (at larger angles, observers become relatively more sensitive to the texture cue^59^). Ensuring similar cue reliabilities (i.e., a 1:1 reliability ratio) gave us the greatest potential to detect the improved performance associated with combination. Specifically, the maximum possible benefit for combining independent cues is a factor of √2 for the case when the two cues have equal reliability; this benefit is smaller when the two cues differ in reliability.

As we were testing the robustness of observers’ perception, we designed the stimulus in the incongruent-cue condition such that one cue was more reliable than the other. To achieve this, we took advantage of the fact that sensitivity to texture-defined slant increases with slant angle^59^. This allowed us to manipulate cue reliability, without changing aspects of the stimuli other than slant (i.e., we did not need to add noise or manipulate contrast, which might complicate comparisons between conditions). Specifically, for the incongruent condition, we combined a smaller disparity slant (*S*_*δ*_ = 20°) with a larger texture slant (*S*_*χ*_ = 50°), yielding a stimulus whose component cue elements differed in reliability (approximately 2:1 ratio). We chose a 2:1 reliability ratio for the incongruent case, as this (i) could be achieved while holding all other stimulus parameters constant between congruent and incongruent conditions (except slant angle), and (ii) was predicted by the model to produce robust behaviour. In addition to the combined conditions, single-cue conditions were included, for each of the slant angles used in the combined stimuli (i.e., *S*_*δ*_ = [40°,20°], *S*_*χ*_ = [40°,50°]). We also included a test stimulus with 0° texture and disparity slant. This was intended to be easily discriminable from the reference stimuli and thus provide a generalized measure of psychophysical performance by capturing the lapse rate of the observers. In addition, we presented six trials with reference stimuli selected at random at the start of each block to refresh observers’ familiarity with the task. Observers were regularly prompted to maintain fixation throughout the experiment.

In the congruent- and single-cue conditions, the test stimuli were defined by congruent and single cues, within a range ±20° of the reference stimulus (40°) over eight evenly spaced steps, i.e., ± [20.0,14.3,8.5,2.8]. For the incongruent-cue condition, the test stimuli were defined by congruent cues, within a range of ± 25° of the midpoint between the slants defined by the incongruent cues of the reference stimulus (35°) over eight evenly spaced steps, i.e., ± [25.0,17.8,10.7,3.6]. For participants who showed high precision in the incongruent condition during the initial familiarization stage, this range was adjusted to ± 14° to more closely assess their sensitivity. As an incongruent test stimulus was compared against consistent-cue reference stimuli, the PSE in the incongruent condition provides an assessment of the perceived shape of the incongruent stimulus in terms of congruent stimuli.

Before brain imaging/stimulation experiments, participants performed a familiarization session in the laboratory. This was used to introduce participants to viewing the stimuli in the stereoscope and ensure they could perform the slant discrimination task.

For the MRS experiment, participants took part in two further sessions. One session was used to acquire MRS measurements inside the MRI scanner while the participants were at rest (i.e., no active task was performed). The other session measured psychophysical performance on the slant discrimination task under the different experimental conditions. The two sessions were separated by 24–48 h and the order of sessions was counterbalanced across participants. For each condition, observers underwent two blocks of 214 trials. Condition order was randomized.

For the tDCS experiments, participants took part in three experimental sessions (sham, anodal or cathodal). Each session was separated by at least 36 h and the order of sessions was counterbalanced across participants. During the initial familiarization session, reference stimuli for the ipsilateral control trials were drawn at random from the pool of reference slants used in the main experiment. During stimulation sessions, the control reference slant was set to that which individual observers could discriminate at 80% performance. Calibration of the eye tracker was performed immediately before the onset of each block in tDCS sessions. Condition order was counterbalanced across simulation sessions and subjects.

### Magnetic resonance spectroscopy

Magnetic resonance scanning was conducted on a 3T Siemens Prisma equipped with a 32-channel head coil. Anatomical T1-weighted images were acquired for spectroscopic voxel placement with an ‘MP-RAGE’ sequence. For detection of GABA, spectra were acquired using a macromolecule-suppressed MEGA-PRESS sequence: echo time = 68 ms, repetition time = 3000 ms; 256 transients of 2048 data points were acquired in 13 min experiment time; a 14.28 ms Gaussian editing pulse was applied at 1.9 (ON) and 7.5 (OFF) p.p.m.; water unsuppressed 16 transients. Water suppression was achieved using variable power with optimized relaxation delays and outer volume suppression. Automated shimming followed by manual shimming was conducted to achieve approximately 12 Hz water linewidth.

Spectra were acquired from three locations; a target (V3B/KO) and two control (V1 and M1) voxels (30 × 30 × 20 mm) (Supplementary Figure 8a). The V3B/KO voxel was positioned in the right hemisphere, adjacent to the median line, and rotated in the sagittal and axial planes so as to align with the posterior surface of the brain, while preventing protrusion from the occipital lobe and limiting inclusion of the ventricles. The V1 voxel was placed medially in the occipital lobe, the lower face aligned with the cerebellar tentorium and positioned so to avoid including the sagittal sinus and to ensure it remained within the occipital lobe. The M1 voxel was defined in the axial plane as being centred on the ‘hand knob’ area of the precentral gyrus and aligned to the upper surface of the brain in the sagittal and coronal planes. These locations are commonly used for defining corresponding target and control voxels in studies linking GABA to cognitive processes^15,60^.

Spectral quantification was conducted with GANNET 2.0^61^ (Baltimore, MD, USA), a MATLAB toolbox designed for analysis of GABA MEGA-PRESS spectra, modified to fit a double-Gaussian to the GABA peak. Individual spectra were frequency and phase corrected before subtracting ‘ON’ and ‘OFF’, resulting in the edited spectrum (Supplementary Figure 8b). The edited GABA peak was modelled off a double-Gaussian (Supplementary Figure 8c) and values of GABA relative to water (GABA/H_2_O; modelled as a mixed Gaussian–Lorentzian) in institutional units were produced. The fitting residual for water and GABA were divided by the amplitude of their fitted peaks to produce normalized measures of uncertainty. The quadratic of these was calculated to produce a combined measure of uncertainty for each measurement^62,63^. This combined fitting residual was relatively low across all participants for all voxel locations, from 3.8% to 9.4% (mean: 6.6% ± 0.2%).

To ensure that variation in GABA concentrations between subjects was not due to differences in overall structural composition within the spectroscopy voxels, we performed a segmentation of voxel content into GM, WM and cerebrospinal fluid (CSF). This was then used to apply a CSF correction^64^ to the GABA/H_2_O measurements with the following equation:

1 $$C_{{\mathrm{tisscorr}}} = \frac{{C_{{\mathrm{meas}}}}}{{f_{{\mathrm{GM}}} + f_{{\mathrm{WM}}}}}$$

where $C_{{\mathrm{tisscorr}}}$ and $C_{{\mathrm{meas}}}$ are the CSF-corrected and -uncorrected GABA concentrations, respectively, and $f_{{\mathrm{GM}}}$ and $f_{{\mathrm{WM}}}$ are the proportion of GM and WM within the voxel. Segmentation was performed using the Statistical Parametric Mapping toolbox for MATLAB (SPM12, http://www.fil.ion.ucl.ac.uk/spm/). The DICOM of the voxel location was used as a mask to calculate the volume of each tissue type (GM, WM and CSF) for both visual and sensorimotor voxels.

### Transcranial direct current stimulation

Direct current stimulation was applied using a pair of conductive rubber electrodes (3 × 3 cm stimulating electrode, 5 × 5 cm reference electrode) held in saline-soaked synthetic sponges and delivered by a battery-driven constant current stimulator (neuroConn, Ilmenau, Germany). For seven participants, functional anatomical scans were used to identify areas V3B/KO in the right hemisphere and then neuronavigational equipment (Brainsight 2, Montreal, Canada) was used to locate the closest point to the centre of mass of this region on subjects’ scalp (Supplementary Figure 9a). The visual cortex electrode was then placed at this location. For the remaining subjects, the average location of this point, relative to positions of the international 10–20 electroencephalography system, was used to place the visual cortex electrode. For all subjects, the reference electrode was placed at position Cz. In the anodal and cathodal conditions, the tDCS current (1 mA) ramped up and down (20 s) before and after continuous application for 20 min. In the sham condition, the current was ramped up then immediately ramped down.

For participants with V3B/KO anatomically localized, FreeSurfer (https://surfer.nmr.mgh.harvard.edu) was used to reconstruct head models from anatomical scans and SimNIBS (http://simnibs.de) used to simulate electric field density resulting from stimulation (Supplementary Figure 9b-d). Simulations indicated that current density was largely unilaterally localized and peaked around V3B/KO.

### Proscriptive integration model

Each primary input (unimodal unit) to the model is specified by its intensity (*A*) and its slant angle in radians (*θ*). The slant receptive field for each primary unit was modelled as a one-dimensional von Mises distribution

2 $$f_{{\mathrm{cue}}}(\theta _{{\mathrm{cue}}}) = A_{{\mathrm{cue}}} \cdot {\mathrm{exp}}\left( {k_{{\mathrm{cue}}}\left[ {\cos \left( {{\mathrm{\pi }} - \theta _{{\mathrm{cue}}\_{\mathrm{pref}}}} \right)} \right]} \right)$$

where *θ*_cue_pref_ indicates cue slant preference. Arbitrarily, *θ*_cue_pref_ takes *n* = 37 evenly distributed values between $- \frac{{\mathrm{\pi }}}{2}$ and $\frac{{\mathrm{\pi }}}{2}$, and the receptive field size, *k*_cue_, was chosen to be 2; producing a slant tuning bandwidth of approximately 10 degrees. The response of each primary unit was assumed to scale linearly with cue intensity, *A*_cue_.

Combination units in the model were generated by drawing input from all possible pairs of unimodal units, as denoted a subscript (*δ* or *χ*), such that there are 37 × 37 = 1396 combination units. Based on previous empirical evidence^27^ we assume that combination units perform a summation of their inputs that increases monotonically, but sublinearly, with stimulus intensity

3 $$E\left( {\theta _\delta ,\theta _\chi } \right) = \sqrt {f_{\mathrm{\delta }}\left( {\theta _\delta } \right) + f_\chi (\theta _\chi )}$$

where *E*(*θ*_*δ*_*,θ*_*χ*_) denotes the activity of the combination unit with disparity slant preference *θ*_*δ*_ and texture slant preference *θ*_*χ*_. The nonlinearity models sublinear response functions of the combination units, which could be mediated by means of synaptic depression or normalization^28^.

The activity of combination units is then passed to a one-dimensional layer of output units. Output units receive input from combination units along the incongruent unimodal preference diagonal with readout weights defined by a cosine, which peaks at unimodal cue preference

4 $$F_i = \mathop {\sum }\limits_{j = - \frac{{\mathrm{\pi }}}{4}}^{\frac{{\mathrm{\pi }}}{4}} E\left( {\theta _{i - j},\theta _{i + j}} \right) \cdot \left( {{\mathrm{cos}}\left[ {4 \cdot j} \right] - c} \right)$$

where *F*_*i*_ denotes the response of the output unit and *c* denotes a temperature offset that models inhibitory dominance of sensory responses^30^. This offset was assumed to be 0.05 for all simulations.

Activity was converted to firing rate by thresholding negative activity values to zero. The height and position of the peak(s) were used to assess estimate reliability and (slant) position^29^. Unimodal responses, for comparison, were generated by setting one of the cue intensities to zero.

For the simulation of Fig. 2c, cue intensities of *A*_*δ*_ = 1 and *A*_*χ*_ = 8 were used to achieve a 1:3 ratio of sensitivity to match previous work^7^. For the simulations in Fig. 4, stimulus intensities of *A*_*δ*_ = *A*_*χ*_ = 1 (single and congruent) and *A*_*δ*_ = 1, *A*_*χ*_ = 4 (incongruent) were used to match the sensitivity ratios engineered for the behavioural stimuli (1:1, congruent; 1:2, incongruent). To simulate variable suppression, an additional parameter (*β*) was used to attenuate the negative readout weights. For each simulation, *β* was set to a value drawn at random from a Gaussian distribution (mean = 0.75, *σ* = 0.1). To simulate individual variability in sensitivity to cues, cue intensity was drawn from a Gaussian distribution (mean = *A, σ* = 0.1).To compare between simulations, we calculated the reliability of the single/combined cue signals relative to one another. For the simulation of Supplementary Figure 1, we systematically varied the variability of the cue intensities from *σ* = [0,0.25].

In the tDCS experiments, we observed that sensitivity for congruent cues in the sham conditions was significantly higher than the maximum bound for fusion (that is, the quadratic sum; offline, *t*_11_ = 4.3, *P* = 0.001, online, *t*_11_ = 3.7, *P* = 0.003). This is likely to be because disparity and texture were not fully isolated in the single-cue conditions, and that these ‘latent’ cues acted to reduce sensitivity to the ‘single’ cue (see Ban et al.^10^ for a discussion of this issue). Thus, to simulate the effects of tDCS with the model (Fig. 5e, f), we first simulated performance in the sham conditions by including a latent cue in single-cue simulations. The intensity of the latent cue was fit using the relative difference between single- and congruent-cue sensitivity (Fig. 5a, c) and held constant for both single-cue simulations. Having fit the model to behaviour in the sham condition, we simulated tDCS by varying two partially free parameters, which independently varied the strength of the positive and negative cosinusoidal readout weights by a factor between zero and one. These parameters model the effects of tDCS on GABAergic (inhibitory) and glutamatergic (excitatory) neurotransmission. We first used the data from the congruent-cue conditions (Fig. 5a, c) to fit these parameters. We then applied these now fixed parameters to the simulation of performance in the incongruent conditions to test their generalizability (Fig. 5b, d).

To simulate perceptual rivalry, the response of the output units *X*_*i*_ is driven by activity of constant strength *F*_*i*_ from the combination layer and produces mutual inhibition (*γ*) through lateral connections with weights defined by a half-wave rectified cosine function. The dynamics of the output units are further defined by slow adaptation (*α*) and stochastic variability (*σ*)

5 $$\tau \frac{{{\rm d}X_i}}{{{\rm d}t}} = F_i - \left( {1 + A_i} \right)X_i + W(0,\sigma ) - \gamma \mathop {\sum }\limits_{j = 1}^N S\left[ {X_j} \right]\left[ {\cos \left( {{\mathrm{\pi }} - \theta _j - \theta _i} \right)} \right]_ +$$

where *S*[*X*_*i*_] denotes a sigmoidal transformation (using a Naka-Rushton function) of the activity of *X*_*i*_, *W* corresponds to Gaussian noise and *A*_*θ*_ represents adaptation

6 $$\tau _A\frac{{{\rm d}A_i}}{{{\rm d}t}} = - A_i + \alpha S[X_i]$$

For the simulation in Fig. 6a, b, cue intensities of *A*_*δ*_* = *1 and *A*_*χ*_* = *1 were used to produce the constant activity in the combination layer *F*(*θ*). Timescales of *τ* = 1 and *τ*_*A*_ = 125 were used to define the temporal dynamics of inhibition and adaptation *γ* = *α* = 7, and the SD of noise was assumed to be *σ* = 0.005.

Maximum likelihood predictions in Figs. 1c, d and 3c were simulated using the following equations:

7 $$\sigma _{\delta \chi } = \sqrt {\frac{{\sigma _\delta ^2\sigma _\chi ^2}}{{\left( {\sigma _\delta ^2 + \sigma _\chi ^2} \right)}}}$$

and

8 $$S_{\delta \chi } = w_\delta S_\delta + w_\chi S_\chi$$

where

9 $$w_\delta = \frac{{r_\delta }}{{r_\delta + r_\chi }},\;w_\chi = \frac{{r_\chi }}{{r_\delta + r_\chi }}$$

Here, *σ* denotes SD of the estimate, *S* denotes slant angle, *r* indicates reliability and *w* denotes weight. Bias is produced by taking the difference between the combined slant estimate (*S*_*δχ*_) and the more reliable single cue slant estimate. SD (*σ*) is converted to sensitivity with the following equation:

10 $$s = \frac{{\sigma ^{ - 1}}}{{\sqrt 2 }}$$

To simulate the normalization model predictions in Figs. 1c, d and 3c, we reproduced the model from ref. ^25^ and the same method of behavioural decoding used in our model to convert firing rate to bias and reliability.

### Data re-analysis

The psychophysical data in Figs. 1c, d, 2c and 6c, d were obtained by extracting the data points from the published papers^7,23^ using WebPlotDigitalizer (http://arohatgi.info/WebPlotDigitizer). The reliability data in Figs. 1c,d and 2c were originally reported normalized to the optimal j.n.d. based on the Gaussian-likelihood model. We transformed this into values relative to single-cue reliability. Similarly, bias was reported as the cue combination direction; this was transformed into bias relative to the more reliable cue. Following this, both bias and reliability data were screened for outliers, binned, averaged and mirror interpolated to show both directions of increasing cue conflict. To screen outliers, we normalized the data using a log transform, removed data points > 3 SD from the mean, then transformed the data back; 1 point was removed from the reliability dataset and 2 from bias. Reliability performance at low cue conflict (bin 1) exceeded quadratic summation predictions of cue fusion based on single-cue reliability (*t*_83_ = 5.6, *P* < 0.001). This is thought to reflect the influence of latent cues present in single-cue stimuli that lead to underestimation of single-cue sensitivity^12^. Thus, to account for this phenomenon, we transformed the reliability data by setting the quadratic prediction as the maximum value of averaged data points.

The bistability data in Fig. 6c, d were originally reported as dual estimates for two slant (perspective/disparity) cues comprising an incongruent stimulus. We transformed these data into a measure of bistability by taking the difference between corresponding estimates. When cues are fused, there will be no difference; however, as bistability ensues, the difference will increase as the observer gains access to each cue.

### Significance testing

To test the significance of behaviour data, we used the repeated measures ANOVA and *t*-test; all tests were two-sided. We first used RM ANOVAs to test for main effects and interactions, we then followed up with *t*-tests as appropriate to determine the precise relationship between conditions. For control/replication experiments, *t*-tests were used to test a priori comparisons. The normality and sphericity assumption was tested with the Shapiro–Wilk test of normality and the Mauchly’s test of sphericity. For the majority of comparisons, no evidence was found for violation of the assumption of normality or sphericity. For comparisons where normality was violated (*n = *2), we applied a transformation to the data to normalize the distribution, then re-tested. For all comparisons, the same pattern of results was found following normalization; thus, for simplicity, we reported the non-transformed comparisons. For comparisons where spherecity was violated (*n = *2), we used the Greenhouse–Giesser corrected *F*-value. To compare the fit of models to behaviour data (Fig. 3c), Bayes factor scores were calculated from *t*-values and reported as odds ratios^65^.

To determine the significance of relationships between brain metabolites and behavioural performance we used the Pearson’s correlation, implemented with a correlation analysis MATLAB toolbox^66^ (https://sourceforge.net/projects/robustcorrtool/). The normality assumption was tested with the Henze-Zirkler test of normality; no evidence was found for assumption violation of the data. The boxplot rule, which relies on the interquartile range^67,68^, was used to reject bivariate outliers; outliers are shown in figures.

### Eye tracking data screening

Before analysis, eye movement data were screened to remove noisy and/or spurious recordings. Owing to the bespoke experimental setup (i.e., recording eye position from behind one-way mirrors in a haploscope) and the time-sensitive nature of brain stimulation (i.e., leaving insufficient time to redo or restart blocks), the eye tracker would occasionally fail to track participants’ eyes for an entire block. Of the 28 blocks (19%) that were omitted from the analysis, 27 had < 1% of data collected. We omitted the remaining block because of (physiologically unlikely) variability in eye position signals that indicated noisy tracking performance. Finally, before averaging trials, we removed points exceeding the radius of the stimulus (4.5°).

### Data availability

The simulation results shown in Figs. 1–6 were generated by code written in MATLAB. The scripts used to implement simulations, and the behavioural and MRS data are available on at 10.17863/CAM.20826.

## Electronic supplementary material

Supplementary Information
