# Supplementary material for: Proscription supports robust perceptual integration by suppression in human visual cortex
Source: Nat Commun. 2018 Apr 17;9:1502. doi: 10.1038/s41467-018-03400-y (PMC5904115; doi:10.1038/s41467-018-03400-y)
Supplement: Supplementary file 1 — Supplementary Information [file 41467_2018_3400_MOESM1_ESM.pdf]

Supplementary Information

Proscription supports robust perceptual integration by suppression in the human visual cortex

Reuben Rideaux and Andrew E. Welchman

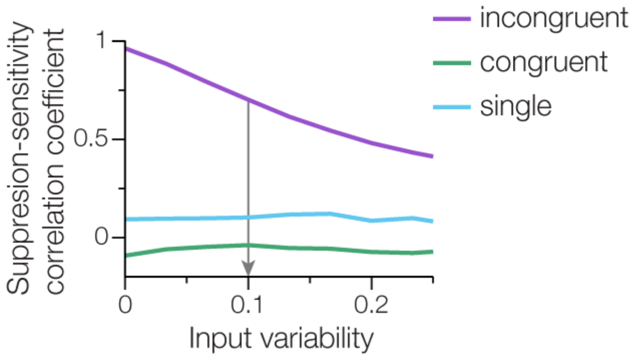

Supplementary Figure 1

Manipulating noise in the model to test the relationship between model suppression and cue sensitivity. In the main text, we used simulations of the Proscriptive integration model to demonstrate a relationship between the amount of suppression in the model, and sensitivity in the incongruent stimulus condition. To demonstrate that this relationship holds in general, we systematically varied the amount of variability in the response of the input layer of the network, and then reassessed the relationship between this parameter and the model's sensitivity. As expected, at low levels of variability the correlation coefficient (rho) between the strength of the model's suppression (negative weights) and simulated cue sensitivity in the incongruent case was close to one. Increasing the variability in the cue inputs led to a drop in the correlation coefficient, but a relationship was still observable. By contrast, the relationship between suppression and sensitivity in the congruent- and single- cue conditions remained around zero for all the different levels of variability assessed. This suggests that the relationships depicted in Figure 4 (main text) hold over a reasonably extensive parameter range. The grey arrow indicates the variability used to produce the simulations shown in the main text.

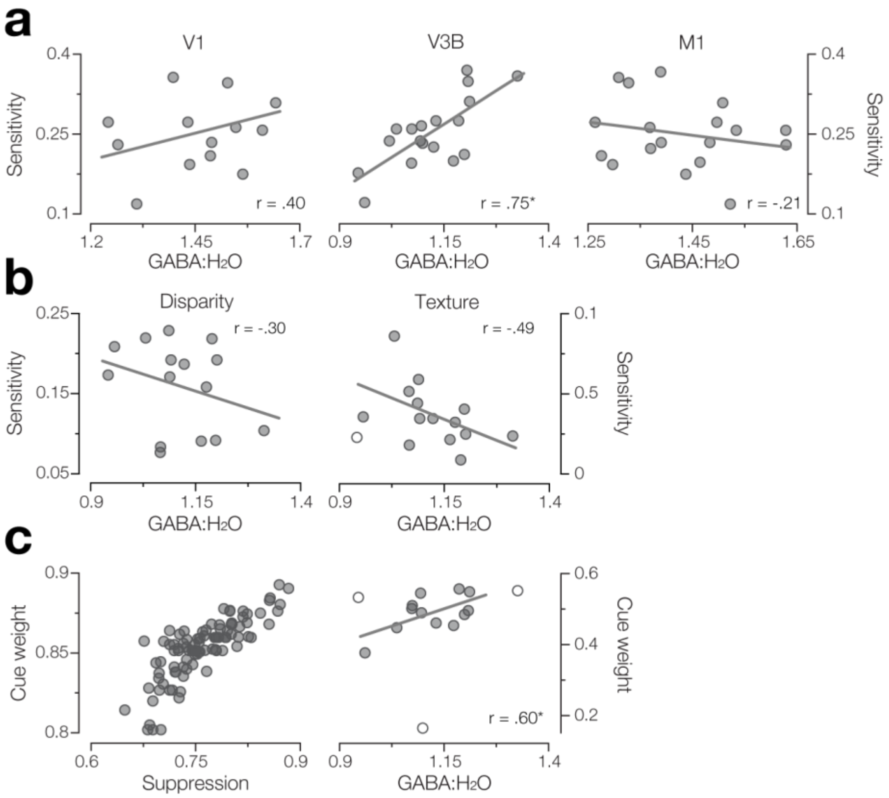

Supplementary Figure 2

Single- and combined incongruent- (disparity/texture) cue sensitivity and cue weights as a function of GABA measured at V3B/KO, V1, and M1 (MRS experiment). We found a significant positive relationship between behavioural sensitivity to incongruent (disparity/texture) cue stimuli and resting GABA concentration measured from a voxel targeting area V3B/KO (**Fig. 4** of the main text). To test whether this relationship was regionally specific to GABA measured at V3B/KO, we assessed the relationship between sensitivity to incongruent cues and GABA measured from two control voxels targeting areas V1 and M1. As shown in (**a**), we found no significant relationship between sensitivity and GABA measured at either control voxel (n = 14, V1: Pearson r=.40, P=.15; n = 18, M1: Pearson r=-.21, P=.39), confirming the regional specificity of the relationship. **Figure 4** (in the main text) also shows that the relationship between sensitivity to incongruent cues and GABA is task specific; that is, we found no relationship between GABA and sensitivity to single- or congruent- cues. The single cue sensitivities presented in **Figure 4** (in the main text) were to slant angles comprising the congruent stimuli. As explained in the Methods, using different slant angles was necessary for the congruent and incongruent stimulus conditions. To further demonstrate that the relationship between incongruent cue sensitivity and GABA was task specific, (**b**) shows that we also found no relationship between GABA and sensitivity to the single cues comprising incongruent stimuli (n = 15, disparity: Pearson r=-.30, P=.26; texture: Pearson r=-.49, P=.08). (**c, left**) Simulations of manipulating the strength of suppression within the proscriptive integration model: reduced inhibition results in more biased estimates for incongruent-cue stimuli. (**c, right**) Same as (**left**) but between human GABA concentration measured at V3B/KO and behavioural cue weight. Each datum represents measures from one participant; bivariate outliers are shown as empty circles. Note that the observed reliable-cue weights (range = [.37, .56]) were smaller than that predicted by the model (range = [.80, .90]). A plausible explanation for this is that for the incongruent-cue reference stimulus, the texture cue was more influenced by a frontoparallel bias, whereas this was not the case for the congruent-cue test stimuli[1]. Thus, while the cues were perceived as incongruent, the magnitude of this difference was reduced as a result of a small frontoparallel bias acting on the texture cue.

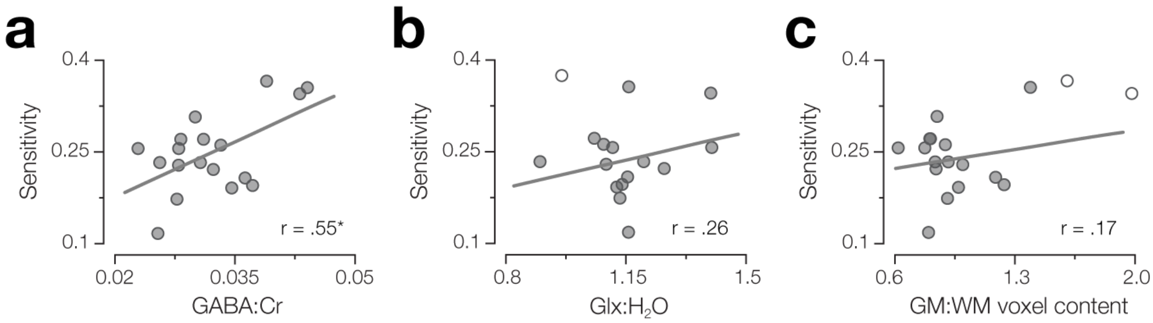

Supplementary Figure 3

Correlations between behavioural sensitivity to incongruent (disparity/texture) cues and GABA referenced to creatine (Cr), and Glx referenced to water (H_2O). We found a significant positive relationship between behavioural sensitivity to incongruent (disparity/texture) cue stimuli and resting GABA concentration measured from a voxel targeting area V3B/KO (**Fig. 4** of the main text). To test whether this relationship could be accounted for by the concentration of the reference metabolite (H_2O), rather than that of GABA, we assessed the relationship between sensitivity to incongruent cues and (**a**) GABA referenced to a different metabolite (Cr) and (**b**) a different metabolite (Glx; glutamate and glutamine) referenced to H_2O. We found that (**a**) the relationship between sensitivity and GABA persisted even when GABA was referenced to a different metabolite (n = 18, Pearson r=.55, P=.02), and (**b**) there was no significant relationship between Glx references to H_2O (n = 17, Pearson r=.26, P=.31), confirming that the significant relationship between incongruent cue sensitivity and GABA shown in **Figure 4** (in the main text) could not be accounted for by the reference metabolite. Note the weaker correlation between sensitivity to incongruent cues and GABA:Cr (r=.55), compared to GABA:H_2O (r=.75). One possible explanation is that the H_2O signal has a better signal to noise ratio and can be modelled more easily[56]; thus, Cr may have been quantified with less accuracy than H_2O, resulting in a weaker correlation. Confirming this, we found that the average normalized fit error (i.e., the standard deviation of the residual over the peak height[2]) for Cr (6.4) was more than twice that for H_2O (2.5; t_{17}=12.81, P<0.001). To test whether the relationship between incongruent cue sensitivity and GABA could be accounted for by differences in the ratio of grey matter (GM) to white matter (WM) voxel content, we assessed the relationship between incongruent cue sensitivity and GM:WM voxel content. We found that (**c**) there was no significant relationship between incongruent cue sensitivity and GM:WM voxel content (n = 16, Pearson r=.17, P=.53). Each datum represents measures from one participant; bivariate outliers are shown as empty circles.

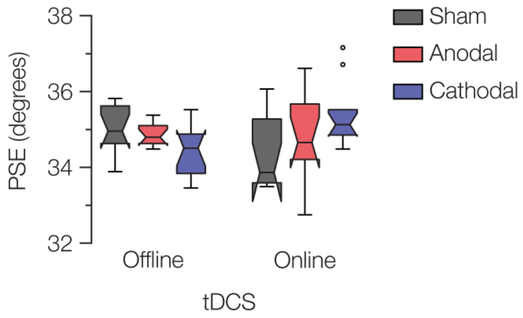

Supplementary Figure 4

Effect of tDCS on bias. Point of subjective equality (PSE) for incongruent cue stimuli for offline and online stimulation. We tested for differences between in the PSE between conditions using a RM ANOVA and found marginally significant effects (offline: $F_{2,22}$=3.22, P=.06; online: $F_{2,22}$=2.72, P=.09). However, the differences were small, and the opposite direction for on- vs. off- line stimulation. Moreover, the largest difference between on- and off- line stimulation is between the sham conditions that provides the control baseline. We therefore interpret these results as serendipitous. The data are presented as boxplots: centre lines indicate the median; box outlines show 25th and 75th percentiles, whiskers indicate 1.5 × the interquartile range, and notches indicate 95% confidence intervals. Extreme values are shown separately (circles).

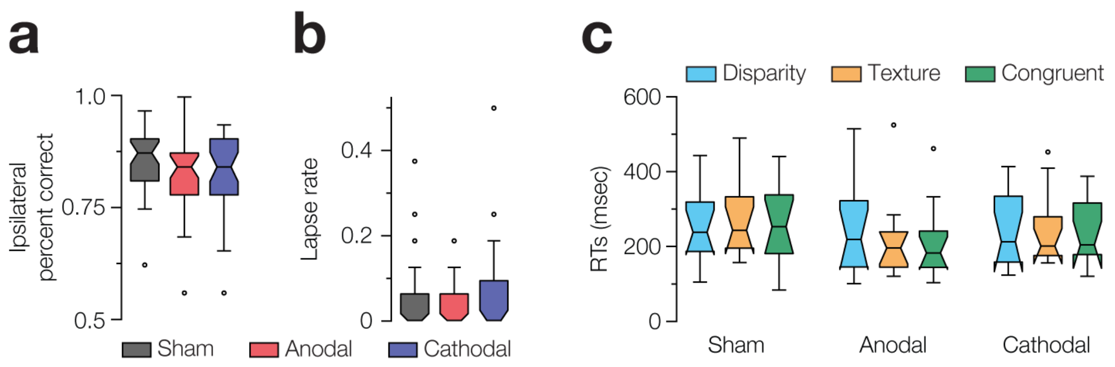

Supplementary Figure 5

Supplementary behavioural measurements for tDCS experiment. (**a**) We assessed performance in the visual hemifield that was unaffected by tDCS (ipsilateral hemisphere) to test whether non-specific effects could explain the tDCS results. We found that performance in the ipsilateral hemisphere did not differ between tDCS conditions (RM ANOVA, $F_{1,22}$=1.19, P=.31), suggesting that the effects seen in the contralateral hemisphere we specific. (**b**) We assessed performance in the contralateral hemisphere (i.e., cortex stimulated by tDCS) for easily discriminable stimuli. We did this to quantify the participants' lapse rates (i.e., the proportion of trials on which they made an error, even though they would have been able to discriminate the stimuli without difficulty). We found that lapse rates did not differ between stimulation conditions ($F_{1,22}$=2.40, P=.11), suggesting that the tDCS effects were not caused by a generalised decrease in attentional state or increased distractibility. (**c**) response times for single- and congruent-cue stimuli presented following 20 min of sham, anodal, and cathodal tDCS. We found no main effect of tDCS type on responses time ($F_{2,22}$=1.54, P=.24), nor an effect of stimulus condition ($F_{2,22}$<1, P=.49). The data are presented as boxplots: centre lines indicate the median; box outlines show 25th and 75th percentiles, whiskers indicate 1.5× the interquartile range, and notches indicate 95% confidence intervals. Extreme values are shown with circles.

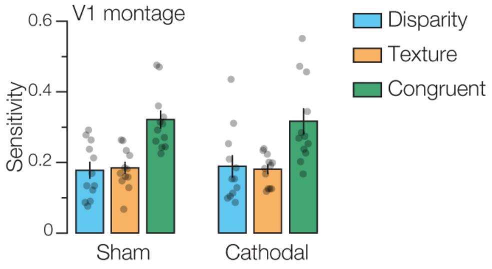

Supplementary Figure 6

Results of tDCS V1-Cz montage control experiment. We assessed whether the tDCS effect observed when we targeted V3B/KO with cathodal stimulation was regionally specific by replicating the experiment using a V1-Cz montage. Semi-transparent black dots indicate individual datum and error bars indicate s.e.m. We found no significant difference between sensitivity to congruent cues following sham and cathodal stimulation of V1 (paired t-test, t_{11}=0.20, P=0.84), suggesting that the tDCS effects observed when targeting V3B/KO were (i) not caused by the reference (Cz) electrode and (ii) were regionally-specific. One could expect perturbing excitability in V1 to disrupt processing of disparity and texture cues by interfering with processing of binocular disparity/orientation tuned units. However, we found no evidence for this; cathodal stimulation did not change single-cue performance. This may suggest that V1 is less susceptible to tDCS (although our electric field modelling suggest this unlikely) or that the particular slanted stimuli we have used are not optimal for revealing behavioural effects from the stimulation of early visual cortex. Nevertheless, the lack of a tDCS effect in V1 provides a useful control in ruling out a generalised, non-specific effect of applying any type of tDCS.

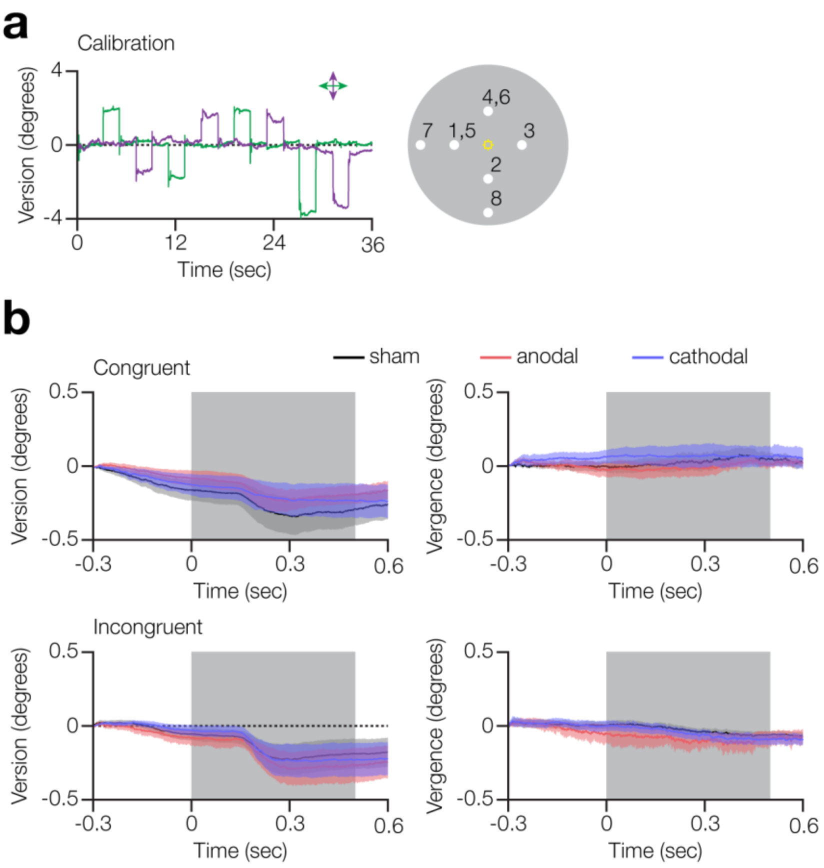

Supplementary Figure 7

Comparison of eye movements across tDCS stimulation conditions. We assessed whether the tDCS effects observed could be explained by differences in eye movements caused by the stimulation. Calibration of the eye tracker was performed immediately prior to the onset of each block in tDCS sessions. (**a**, left) Eye movements during the calibration period from a representative observer; (**a**, right) target locations and their temporal order. (**b**) Average version and vergence eye movements for congruent- and incongruent- cue stimuli following sham, anodal, and cathodal stimulation. The grey region of the plot indicates the period of stimulus presentation; shaded regions indicate ±1 s.e.m. To quantify any change of horizontal vergence and version position that might take place during a trial, we fit a line to the eye vergence/version data of individual trials during the 500 ms window corresponding to stimulus presentation. We thereby quantified vergence/version changes on each trial in terms of the gradient (*β*) of the best fitting (least-squares) linear model to the data. Thereafter we compared the gradient terms between stimulation conditions, we found no differences for vergence (congruent: $F_{2,26}$=1.23, P=.31, incongruent: $F_{2,26}$<1, P=.53) or version (congruent: $F_{2,26}$<1, P=.89, incongruent: $F_{2,26}$<1, P=.88). These data suggest that changes in vergence/version do not provide an account for the tDCS effects observed in our experiments.

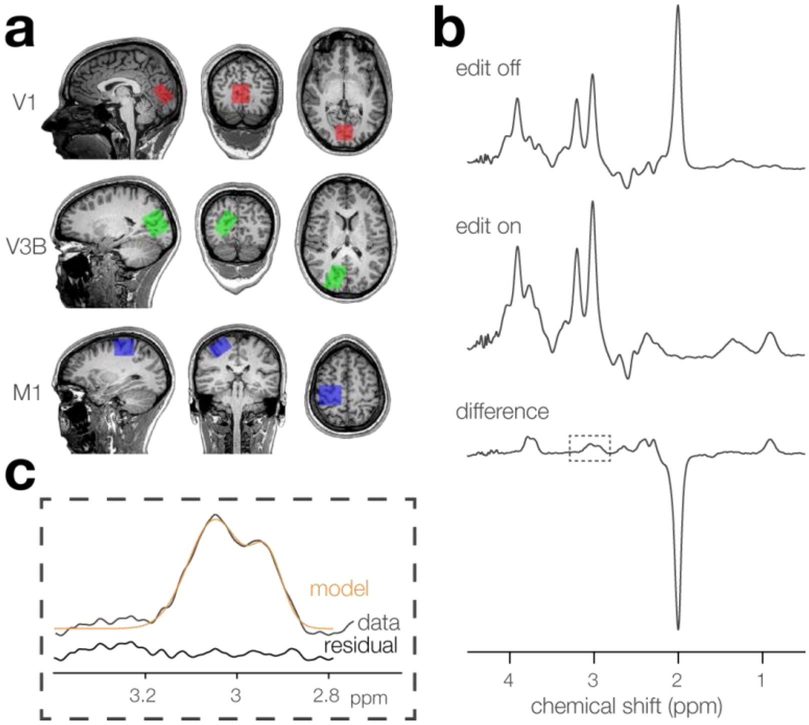

Supplementary Figure 8

Example voxel placement, MRS spectra and GABA peak fitting (MRS experiment). Spectra were acquired from one target (V3B/KO, green) location and two control (V1, red; M1, blue) locations. (**a**) Voxel locations for the three locations shown on MRI images from one participant. Individual spectra were frequency and phase corrected before subtracting ON and OFF, resulting in an edited spectrum. (**b**) Example edit off, edit on, and difference spectra; the dotted-black rectangle on the difference spectrum shows the location of GABA. To quantify the concentration of GABA, a double-Gaussian was fit to the peaks at 3 ppm. (**c**) The double-Gaussian model fit to the GABA peak in (**b**); the data, model fit, and residual are indicated by the grey, yellow, and black lines, respectively.

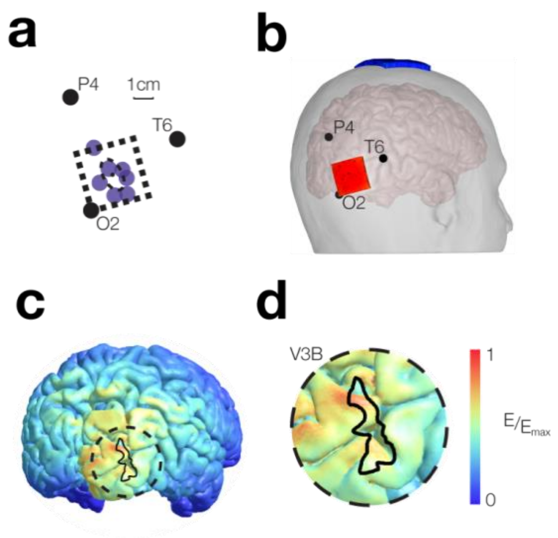

Supplementary Figure 9

tDCS montage and electric field density simulations. (**a**) Transcranial V3B/KO locations identified with neuronavigation: purple circles indicate the location across participants, relative to (P4, O2 & T6) 10-20 EEG positions. The small black dot shows the average location, the dashed ellipse indicates the standard error, and the dashed square delineates the patch placement. (**b**) Montage used in the experiment: target electrode over V3B/KO, reference over Cz. We used FreeSurfer (https://surfer.nmr.mgh.harvard.edu) to reconstruct head models from anatomical scans and SimNIBS (http://simnibs.de) to simulate electric field density resulting from stimulation. (**c**) Representative simulation of electric field density resulting from stimulation. (**d**) Close-up of (**c**) simulated electric field density centred on functionally localized area V3B/KO (outlined in black; centered on coordinates [x=93, y=211, z=128] in Talairach space). Heat maps in (**c**) and (**d**) indicate electric field strength from 0 (blue) to maximum (red).

SUPPLEMENTARY REFERENCES

1. Saunders, J. A. & Chen, Z. Perceptual biases and cue weighting in perception of 3D slant from texture and stereo information. *J. Vis.* **15,** 14–14 (2015).

2. Edden, R. A. E., Puts, N. A. J., Harris, A. D., Barker, P. B. & Evans, C. J. Gannet: A batch-processing tool for the quantitative analysis of gamma-aminobutyric acid-edited MR spectroscopy spectra. *J. Magn. Reson. Imaging* **40,** 1445–1452 (2014).
